# Diagnosis and management of infected arthroplasty

**DOI:** 10.1051/sicotj/2021054

**Published:** 2021-11-01

**Authors:** Tejbir S. Pannu, Jesus M. Villa, Carlos A. Higuera

**Affiliations:** Levitetz Department of Orthopaedic Surgery, Cleveland Clinic Florida 2950 Cleveland Clinic Blvd. Weston 33331 FL USA

**Keywords:** PJI, Periprosthetic joint infection, Infected arthroplasty, Diagnosis of PJI, Treatment of PJI

## Abstract

Periprosthetic joint infection (PJI) is one of the most dreadful complications after THA and TKA. Though prevention is of utmost importance in PJI management, the last decade has seen many remarkable developments in PJI diagnosis, including the introduction of several standardized PJI diagnostic definitions and biomarkers. Depending on the specific clinical situation, a myriad of treatment options for PJI are offered. Our review aims to summarize the pertinent information on PJI diagnosis and synthesize literature on the different treatment methods currently used in clinical practice. One of the most accepted PJI diagnostic definitions was developed by the Musculoskeletal Infection Society (MSIS) in 2011, later modified in the 2013 International Consensus Meeting (ICM). After promising results from studies, alpha-defensins and D-dimer were recently incorporated into the 2018 ICM PJI definition. The management choices for PJI include irrigation and debridement (DAIR), one-stage exchange arthroplasty, or two-stage exchange arthroplasty, to name a few. While two-stage revision has traditionally been the treatment of choice in the United States, there has been a growing body of evidence framing one-stage revision as a comparable choice. One-stage revision should be offered in patients meeting strict selection criteria: no sinus tract, proper soft tissue available for wound closure, appropriate bone stock, a favorable identifiable organism with encouraging antibiotic sensitivities (for cement and oral suppression later), and robust immunological status. DAIR can be considered in case of early infections with sensitive infecting organisms. Patients with multiple unsuccessful revisions or those who refuse further surgical intervention for PJI can be offered antibiotic suppression. If nothing seems to work, salvage procedures (resection arthroplasty and arthrodesis) are available as a last resort. Further research is encouraged to improve on diagnostic capabilities and develop evidence on the best treatment of choice for PJI.

## Introduction

Total joint arthroplasty is one of the most common orthopedic procedures performed in the United States. More than a million patients undergo total hip and knee arthroplasties (THA and TKA) per year [1]. The statistics are predicted to surge in the coming years [2], and with that, the complications associated with joint replacements. Periprosthetic joint infection (PJI) is one of the most dreadful complications after THA and TKA, its incidence roughly varies between 1% and 2% [3, 4]. From the standpoint of quality of life, infected arthroplasty results in a significant decrease in affected patients when compared to the general population [5]. Even though prevention is of utmost importance in the management of PJI, the first step to solve a problem is always “identification,” and the last decade has seen many remarkable developments in the diagnosis of PJI. These developments include the introduction of several standardized PJI diagnostic definitions from the national scientific societies and organizations [6–9], and later, biomarkers and better culture techniques [10] to improve the diagnostic capabilities. The second step after identification is “solution,” and depending on the specific clinical situation, treatment options for PJI include irrigation and debridement, one-stage exchange arthroplasty, or two-stage exchange arthroplasty, just to name a few. While two-stage revision has traditionally been the treatment of choice in the United States [11], there has been a growing body of evidence framing one-stage revision as a comparable choice [12–14].

Despite the best efforts and advances about the diagnosis and treatment of the infected arthroplasty, there is no gold standard diagnostic test or treatment. The evidence on these subjects is developing incessantly, the evidence is growing. Thus, in our review, our objective is to summarize the pertinent information on the diagnosis of PJI and synthesize literature on the different available treatment methods currently used in clinical practice.

## Diagnosis

Over the years, multiple definitions and markers have been proposed to define periprosthetic joint infection. Hereafter, we discuss the most relevant proposed PJI diagnostic criteria and biomarkers used for that purpose.

One of the first and most accepted PJI diagnostic definitions was developed by the Musculoskeletal Infection Society (MSIS) in 2011 [6], which was later modified in the 2013 International Consensus Meeting (ICM) [7]. The application of these criteria require a physical examination, preoperative serum, synovial laboratory tests including cultures, and intraoperative frozen sections with histology. Based on this consensus-based PJI definition (Table 1), the presence of two positive periprosthetic cultures with phenotypically identical organisms, or a sinus tract communicating with the joint, or positive three out of five minor criteria are considered diagnostic of PJI. The minor criteria include: (i) erythrocyte sedimentation rate (ESR) more than 30 mm/h and C-reactive protein (CRP) more than 10 mg/L, (ii) more than 3000 WBCs/μL/positive leukocyte esterase, (iii) polymorphonuclear (PMN)% more than 80%, (iv) one positive culture with the identified organism, and (v) more than five neutrophils per high power field (HPF) in five HPFs on the histologic analysis [7]. It needs to be acknowledged that even if three criteria are not met, PJI might still be existent. In addition, each batch of 3–5 tissue/fluid samples needs to be taken with a different set of sterile instruments. When the required number of criteria are not met, some conclusions could be drawn from the virulence of cultured organisms (if any). If it is a low virulence organism (Corynebacterium, Cutibacterium acnes, or coagulase-negative Staphylococcus), this might not signify PJI. However, if it is a single virulent organism (*Staphylococcus aureus*), this may characterize infection [7]. The 2013 ICM definition is the most commonly used PJI definition. Nevertheless, this definition has shown poor performance for diagnosing infection in some circumstances [15–20]. For instance, in patients with metal-on-metal implants, this criterion is associated with high false-positive results [15–17]. In the setting of two-stage revision, the 2013 ICM definition has shown low sensitivity (0–25%) to confirm infection control and predict failure of reimplantation [18–20]. Thus, this definition appears to have limited screening capability for infection at the time of reimplantation.

**Table 1:** 2013 International Consensus Meeting (ICM) modified Musculoskeletal Infection Society (MSIS) consensus-based criteria for the diagnosis of periprosthetic joint infection (PJI).

Diagnosis of PJI (knees and hips)
1. Two positive periprosthetic cultures with phenotypically identical organisms, or
2. A sinus tract communicates with the joint, or
3. Having three of the following minor criteria: Elevated serum C-reactive protein (CRP): more than 10 mg/L AND erythrocyte sedimentation rate (ESR): more than 30 mm/h Elevated synovial fluid white blood cell (WBC) count: more than 3000 cells/μL OR ++ change on leukocyte esterase test strip Elevated synovial fluid polymorphonuclear neutrophil percentage (PMN%) more than 80% Positive histology of periprosthetic tissue One positive culture

C-reactive protein (CRP); erythrocyte sedimentation rate (ESR).

Another set of clinical practice guidelines were proposed by the Infectious Diseases Society of America (IDSA) for PJI diagnosis [8]. According to these recommendations, when a sinus tract communicating with the prosthesis or purulence without any other known cause is present, there is a definitive PJI diagnosis. Further, two or more pre-/intraoperative cultures of the same organism were also deemed as definitive PJI. Similar to the aforementioned ICM definition, a single virulent organism (*S. aureus*) might represent PJI. Any signs of acute inflammation on histopathologic examination of tissue samples were considered indicative of infection. This set of recommendations have not been investigated in their ability to diagnose PJI as such [8].

Due to low sensitivity to rule out infection using traditional WBC-count and PMN% thresholds (3000/μL and 80%, respectively) in the widely used 2013 ICM definition, several thresholds of WBC-count and PMN% have also been tested to determine improvements in diagnostic accuracy. New thresholds of these markers to detect infection (ranges: WBC-count = 970/μL to 4450/μL, and PMN% = 56–80%) have been proposed with variable sensitivities (ranges: WBC-count = 39.1–92.9%; PMN% = 67–76%) and specificities (ranges: WBC-count = 10–76%; PMN% = 56–80%) [21–26].

To improve the PJI diagnostic accuracy, several serum and synovial tests/biomarkers have been investigated [10]. Among the synovial markers, alpha-defensins, CRP, leukocyte esterase, IL-6, IL-1β, and IL-17 have been demonstrated to have high odds ratios in patients with PJI [10]. Out of all these, alpha-defensin has the best ability to diagnose PJI, with 748.3 times more odds of positive test results in patients with infection versus those without it [10]. Several meta-analyses have pooled data from multiple studies to assess the predictive ability of alpha-defensin and leukocyte esterase. The results from three meta-analyses on this subject demonstrated alpha-defensin and leukocyte esterase to pooled PJI diagnostic sensitivities/specificities ranging from 87% to 100%/96% to 97% and 81% to 90%/96% to 97%, respectively [27–29]. Overall, both synovial markers seem to have high PJI diagnostic accuracy. Recently, alpha-defensins has been tested against the 2013 ICM and IDSA definitions of PJI. This synovial marker exhibited high specificity of more than 95% (better than both definitions) and sensitivity of 84% (better than 2013 ICM) and IDSA (67%), showing potential as an affirmative rather than a screening test [30]. D-Dimer, a serum-marker, is promising for diagnosing PJI in an initial investigation. Serum D-Dimer threshold of 850 ng/mL demonstrated better sensitivity (89%) and specificity (93%) than ESR and CRP (ESR: 73%/79% and CRP: 78%/80%, respectively) [31]. Notwithstanding, conflicting results have been found with many studies regarding its performance in diagnosing PJI [32, 33].

The search for an actual “gold standard” definition/criteria for PJI diagnosis led to the development of the new PJI diagnostic criteria [9]. With the incorporation of promising diagnostic tests such as synovial alpha-defensin and serum D-Dimer, the new 2018 ICM definition of PJI has been recently proposed (Table 2) [9]. While most previous PJI definitions were consensus-based, this recent definition was evidence-based with a weight-adjusted scoring system for underlying criteria/biomarkers and internal and external validation of the definition in a series of patients. Under this PJI diagnostic definition, two positive cultures of the same organism or a sinus tract communicating with the joint or exposure of the prosthesis are deemed as definitive PJI diagnoses (major criteria). “Minor criteria” includes both preoperative and intraoperative tests/criteria, with a scoring system to define PJI. A total score ≥ 6 represented PJI, a score of 2–5 is indecisive and needs the consideration of intraoperative criteria to confirm/refute infection diagnosis, and a score of 0 or 1 does not define PJI (Table 2). This new definition revealed a sensitivity and specificity of 97.7% and 99.5, respectively. When compared to the 2013 ICM definition, the 2018 definition showed much higher sensitivity (2018 ICM [97.7%] vs. 2013 ICM [86.9%]), even though both had similar specificity [9].

**Table 2:** The new evidence-based 2018 International Consensus Meeting (ICM) definition for diagnosing periprosthetic joint infection (knee and hips).

Major criteria		
1. Two + cultures (same microorganism)	Interpretation	
2. Sinus tract communicating with the joint or if joint prosthesis is visualized	At least one of these: Infected	
Minor criteria		
A. Preoperative diagnosis	Score	Interpretation
*Serum*		
1. ↑CRP (more than 1 mg/dL) OR D-Dimer (more than 860 ng/mL)	2	≥6: Infected
2. ↑ESR (more than 30 mm/h)	1	2–5: Possibly infected
*Synovial*		0–1: Not Infected
1. ↑Synovial WBC count (more than 3000 cells/μL) or LE ++	3	
2. +Alpha-defensin (signal-to-cut-off ratio > 1)	3	
3. ↑Synovial PMN (%) (more than 80%)	2	
4. ↑Synovial CRP (more than 6.9 mg/L)	1	
B. Intraoperative diagnosis		≥6: Infected
1. Preoperative score	–	4–5: Inconclusive
2. +Histology	3	≤3: Not Infected
3. +Purulence	3	
4. One + Culture	2	

C-reactive protein (CRP); erythrocyte sedimentation rate (ESR); positive (+); ↑: increase; polymorphonuclear neutrophils (PMN).

Recognizing the fact that the 2018 ICM definition of PJI was supported by only 68% of meeting delegates and also not endorsed by MSIS, yet another most recent PJI definition has been recently proposed by the European Bone and Joint Infection Society (EBJIS) (Table 3) [34]. The EBJIS definition categorizes scenarios into three: infection unlikely, infection likely, and infection confirmed. The details on these categories and their underlying tests have been presented in Table 3. Based on their significance, different tests and their values have been grouped differently [34]. For instance, for “infection unlikely” result, there shall be no positive suggestive or confirmatory test, and for “infection confirmed”, even one positive test under this category can define the presence of infection. The middle category of “infection likely” covers all likelihood situations of infection and when additional investigations should be considered. Of note, even in the case of multiple positive test results under this category, a confirmatory test under “infection confirmed” category is required to establish the diagnosis of PJI [34]. While this new EBJIS is backed by the selective arthroplasty community, its performance remains to be widely investigated in PJI patient populations. It is important to note that the evolution of PJI definitions from 2011, 2013 MSIS to 2018 ICM to the most recent EBJIS definition might confuse surgeons while choosing the right definition in their clinical practice. At the time of this writing, the 2013 MSIS definition is still the most commonly used among surgeons until robust evidence emanates on the superiority of the newly proposed definitions. Each definition has its limitations, and the researchers have tried to improve upon the diagnostic performance over the years.

**Table 3:** The European Bone and Joint Infection Society (EBJIS) definition for diagnosing periprosthetic joint infection (knee and hips).

Infection	Unlikely	Likely	Confirmed
Clinical/blood			
Clinical features	Implant dysfunction due to alternative cause (fracture, implant breakage, malposition, tumor).	Loosening on radiological analysis (≤5 years after implantation). History of wound healing issues. Recent fever or bacteremia. Periprosthetic purulence.	Sinus tract communicating to the joint or when prosthesis can be visualized.
CRP		More than 10 mg/L	
Synovial fluid			
Leukocyte count (cells/μL)	≤1500	>1500	>3000
PMN%	≤65%	>65%	>80%
Alpha-defensin			Positive
Microbiology			
Culture – aspirated fluid		Positive	
Culture – intraoperative	Negative	One positive	≥Two positives (same organism)
Sonication (CFU/mL)	No growth	>1 CFU/mL (any organism)	>50 CFU/mL (any organism)
Histology – High-power field (400×)	Negative	≥5 neutrophils/HPF	≥5 in ≥HPFs
			Visible organisms
Nuclear imaging	Negative 3-phase isotope bone scan	Positive white blood cell scintigraphy	

C-reactive protein (CRP); erythrocyte sedimentation rate (ESR); CFU: colony forming unit; polymorphonuclear neutrophils (PMN).

One of the oldest diagnostic tests is also important: frozen section (operator-dependent test), which is a minor criterion under various PJI definitions. The performance of intraoperative frozen section in PJI diagnosis has been recently investigated in a meta-analysis of 26 studies with a total of 3269 patients [35]. The frozen section (>5 PMNs per HPF) demonstrated 52.6 times more odds of getting a positive test result in cases with PJI than in those with no PJI. The testing of frozen section against the 2013 ICM criteria showed high specificity of 98.8% and moderate sensitivity of 73.7%, supporting its role as a rule-in or confirmatory test [36].

## Treatment (Figure 1)

### DAIR

DAIR is an acronym for debridement, antibiotics, and implant retention. While both two-stage and one-stage revision involve the removal and implantation of a new prosthesis, DAIR involves the retention of the implant secured to the bone, with the removal of just polyethylene (PE) insert/liner and all the remaining modular parts followed by a thorough radical debridement, and re-insertion of a new insert/liner. This approach is simple, preserves bone stock, reduces costs, and decreases morbidity compared to the implant exchange revisions [37]. However, the success rate in the setting of PJI is variable. Timing and causing organisms of the infection to seem to impact the outcomes achieved with DAIR. Di Benedetto et al. found this procedure more useful in the setting of early acute or acute, delayed PJIs [38]. With respect to the profile of the infecting organism, DAIR is considered more suitable in the case of early and sensitive Staphylococcal infections (<7 days symptoms or drainage) [39]. However, in PJI, where methicillin-resistant *Staphylococcus aureus* is the causative organism, this treatment method has been shown to fail in 84% of the cases [40]. While DAIR should not be performed for PJI with organisms like MRSA, delay in treatment until culture results (48 h or more) is debatable. A recent investigation compared the success with DAIR between two timelines: more than two days of symptom onset until cultures versus within two days of symptom onset. DAIR was indicated in these patients based on decision-making algorithms [41]. Bedair et al. did not find any differences and determined DAIR to be successful in only 57% of non-Staphylococcal PJI cases [41]. They found the infecting organism to be a significant predictor of treatment success. While the overall success of this procedure ranges from 20% to 75% in knee PJIs [42–44], 80% success rate has been reported in hip PJIs by Rava et al. [45]. The use of systemic antibiotics and long-term chronic oral antibiotic suppression in patients undergoing this procedure has been shown to reduce the failure rates [46]. In the literature, several scoring systems (KLIC and CRIME80 score) have been proposed to predict the success of DAIR in late acute PJIs [47, 48]. These scores are based on preoperative risk factors, including comorbidities, inflammatory markers, type of prosthesis, etc. KLIC-score includes five preoperative risk factors and stands for Kidney (score = 2), Liver (1.5), Index surgery (1.5), Cemented prosthesis (2), and C-reactive protein > 115 mg/L (2.5) [47]. In the study by Tornero et al., patients with KLIC-scores of two and seven had failure rates of 4.5% and 100%, respectively. Beginning at a KLIC score of 4, DAIR success rate is <45% [48]. CRIME80 score is used for hematogenous infections covering seven preoperative risk factors and stands for chronic obstructive pulmonary disease (COPD) (score = 2), CRP > 150 mg/L (1), rheumatoid arthritis (RA) (3), index surgery (following fractures) (3), male (1), polyethylene exchange (−1), and 80 years of age (2). There have been mixed results on their performance. A CRIME-80 score ≥ 3 reduces the probability of surgical success to <40% [48].

**Figure 1:**
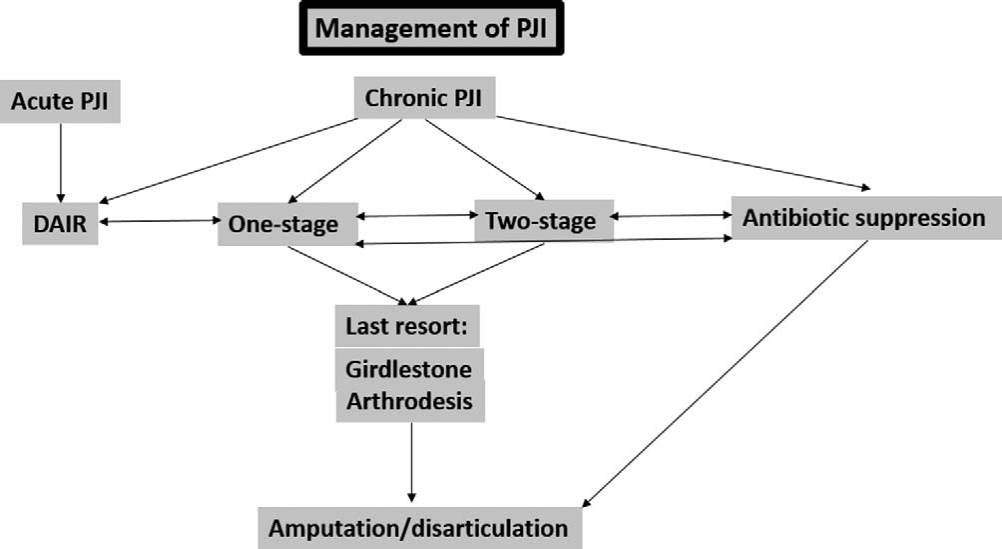
An algorithm of the management of periprosthetic joint infection (PJI) – acute and chronic.

### One-stage revision arthroplasty

In the setting of PJI, even though two-stage revision is the treatment of choice in the United States [11], this is not the only option. The literature reports a mortality rate of 26% at five years in patients undergoing two surgeries in a two-stage revision [49]. This implies that surgeons must consider a single surgery (one-stage revision) as the indicated procedure in a selected group of patients. One stage exchange arthroplasty involves removing the implants, performing a thorough radical debridement, mechanical and chemical disruption of the biofilm, and placing new implants all during the same surgical episode.

While multiple investigations have demonstrated success rates ranging from 75% to 100% for the two-stage treatment [50–54], it was determined in the 2013 ICM that the actual success rate (no reinfection, no reoperation/surgical intervention and no adverse mechanical outcome) of this treatment approach was just 65% [55]. On top of that, recent studies have revealed comparable reinfection rates in patients undergoing one-versus two-stage revision with rather a drift towards superior functional clinical outcomes in those who had one-stage procedures [14, 56]. Haddad et al. compared the one-stage and two-stage revision treatments. For 102 chronic knee PJI patients, the authors devised strict selection criteria to indicate one-stage revision. The selection criteria were as follows: minimal-moderate bone loss, absence of immunocompromising conditions, healthy soft tissues (extremity grade 1), and a known organism with known sensitivities for which appropriate antibiotics were available for addition to the cement at the time of reimplantation, and after that, for chronic oral suppression. Not a single one-stage patient developed a recurrent infection, but five two-stage patients had reinfection at a minimum follow-up of three years. Furthermore, one-stage patients had superior knee society scores as compared to those who underwent two-stage treatment [14].

For one-stage revision THA, Singer et al. demonstrated a success rate of 95% on the exclusion of those hips that cultured MRSA [57]. In a recent report, the success rate of 100% for PJI has been demonstrated in the case of one-stage hip and knee revisions [58]. Thus, one-stage revision should be considered in patients who meet strict selection criteria: no sinus tract, proper soft tissue available for wound closure, appropriate bone stock, a favorable identifiable organism with encouraging antibiotic sensitivities (for cement and oral suppression later), and robust immunological status. Since the studies show that the success of repeated revision arthroplasties goes down along the curve to 60%, choosing the right procedure at the time of presentation is the key [14, 56, 59].

### Two-stage exchange arthroplasty

Two-stage revision is the treatment of choice for chronic PJI in the United States [11]. This procedure involves surgery in two stages with a time interval between the two. The first stage of two-stage revision comprises the removal of the prosthesis, a thorough irrigation, and debridement, mechanical and chemical disruption of the biofilm, subsequently followed by the insertion of antibiotic-eluding cement spacer (static or articulating) with a new set of clean drapes and instruments. The role of spacers is to maintain the joint space and motion (articulating spacer) while PJI is being treated with antibiotic therapy and until the infection control and subsequent final reimplantation [60]. Overall, articulating spacers have been known to result in a better range of motion and outcomes than the static spacer. Nevertheless, in cases with ligamentous instability, widespread bone loss, or compromised soft tissue coverage, static spacers are a better choice to provide stability, reduced soft tissue tension, and reduced bone loss rate [60]. In addition to preoperative work-up, intraoperative tissue samples are recommended for cultures. It is paramount to give prophylactic antibiotics to the patient before incision, as withholding antibiotics before collecting culture samples has been shown not to increase the likelihood of obtaining positive cultures [61, 62]. After confirmation of infection control, the second stage of two-stage revision involves removing the cement spacer, followed by reimplantation of the new prosthesis. In the time interval between the two stages, antibiotic therapy is indicated for approximately six weeks. In the setting of PJI, mostly parenteral antibiotic therapy is traditionally used [63]. However, studies have shown that transition to oral antibiotic therapy after an initial short course of intravenous antibiotic therapy is effective. In the study by Ascione et al. on 122 PJI patients who underwent two-stage revision, 52 patients received intravenous antibiotic therapy in the entire interim period before reimplantation and 70 patients were administered two weeks of intravenous antibiotic therapy with transition to oral antibiotic therapy in the remaining interim period [64]. They found that the use of oral antibiotics (Odds ratio = 5.3; *p* = 0.02) was significantly associated with treatment success of two-stage revision [64]. An investigation by Darley et al. in the setting of hip PJIs also found that an early switch from intravenous (for 10–14 days) to oral antibiotics (6–8 weeks) is a viable option [65]. An antibiotic holiday of two weeks is customary before reimplantation, but the evidence is limited to support the need or duration of this period (agreed by 92% delegates in the ICM) [7]. The primary reason is to measure serum ESR, CRP and test synovial fluid for indicators of infection such as WBC counts, PMN%, and to perform cultures [66]. This time-off antibiotics before reimplantation to identify persistent infection has been controversial. A study by Tan et al. on 409 two-stage revisions that were performed after the first stage with no interim procedures with a treatment success rate of 84.4% demonstrated no changes in treatment failure rates with variable duration of antibiotic-free periods (one week, two weeks, four weeks) before reimplantation [67]. On the contrary, on comparing the reimplantations in which continuous antibiotic therapy was instituted versus those in which antibiotic holiday of two weeks was implemented, Ascione et al. determined continuous antibiotic treatment in the interim between the first and second stage as a significant predictor of treatment success (Odds ratio = 3.32; *p* = 0.02) [68]. Furthermore, they found that compared to the patients who had an antibiotic-free interval before reimplantation, the continuous antibiotic treatment led to a significantly higher treatment success rate in immunocompromised patients (46 patients vs. 31 patients; *p* = 0.02) [68]. In general, choosing the antibiotic-free interval or not is mostly based on the surgeon’s experience and preference with no one definitive answer.

The importance of the confirmation of infection control before reimplantation cannot be overemphasized. When it comes to accurate criteria or definition to rule out infection at the time of reimplantation, we run out of options due to the low sensitivity (0–25%) of the most accepted 2013 ICM criteria. Due to high specificity, the combination of 2013 ICM criteria and frozen sections are often recommended during reimplantation [18, 69]. Several biomarkers such as alpha defensin have been investigated to confirm infection control. Since alpha defensin has not been validated for diagnosing PJI in patients with cement spacers, its reliability either alone or together with results of preoperative aspiration is questionable to confirm control of infection before reimplantation [70, 71]. The importance of synovial fluid aspiration before reimplantation has been controversial. The chief reason being that these patients on cement spacers (after first stage) have smaller amount of synovial fluid and lower WBC thresholds as compared to standard total joint prosthesis [72]. Thus, not even a single definition/test/criterion is conclusive of infection status to decide the right time of reimplantation in two-stage revision. Clinical examination and varying test combinations, mainly cultures, are used for surgical decision-making at the time of reimplantation.

Two-stage revision might be more appropriate for patients infected with more virulent and/resistant bacteria, soft tissue deficiencies, and reduced bone stock [57]. The bacteria which are difficult to treat (Methicillin-resistant *Staphylococcus aureus* (MRSA), *Enterococcus*, or gram-negative bacteria) are associated with a lower rate of infection control after surgery, significantly predictive of surgical failure [73]. Disadvantages of two-stage arthroplasty include increased cost, cement spacer morbidity with decreased quality of life, joint contractures, disuse osteopenia, muscle atrophy, and increased mortality [74–77]. Such adverse outcomes should always be discussed with the patient before embarking on such treatment.

In the clinical set-up, there are patients who are either frail as determined by the surgeon or do not want to proceed with the second stage, in these patients, retention of the spacer is an option. In a recent case series, 35 out of 94 patients retained the spacer for more than one year, and 31 out of these 35 patients did not develop a recurrent infection or need any additional surgical intervention at a follow-up of three years [78].

### Antibiotic suppression alone

Antibiotic suppression is not a first-line treatment and is offered to PJI patients who have been through multiple unsuccessful revisions and is reserved for those who cannot tolerate another procedure or those who refuse further surgical intervention. Extended oral antibiotics were recently shown to be associated with a significant reduction in failure rate after DAIR (hazard ratio: 2.47) compared to only intravenous antibiotics [79]. The recommended duration of antibiotic administration in patients is variable, but intake beyond one year in knee PJIs was not found to have a significant advantage versus less than one year by Shah et al. [61]. The success of oral antibiotic suppression is also dictated by the type of infecting organism. Leijtens et al. studied 23 patients with hip PJI treated with antibiotic suppressive therapy for a mean duration of 38 months and found the overall failure rate to be 43.5% [80]. In their patient cohort, PJIs with *Staphylococcus aureus* had a failure rate of 80% compared to 33% in the case of PJI with any other causative organism [80]. In another investigation, Siqueira et al. documented a marked increase in infection-free survival (68.5%) with the use of chronic antibiotic suppression (minimum six months) versus lack thereof (41.1%) in hip or knee PJI patients who underwent irrigation and debridement with polyethylene exchange or two-stage revision [46]. Patients who had irrigation and debridement with polyethylene exchange (64.7%) and those who had PJI caused by *S. aureus* (57.4%) showed the highest increase in survival with chronic oral antibiotic suppression [46]. With patients put on prolonged antibiotic suppressive therapy for PJI, Prendki et al. conducted a nationwide database study to ascertain the clinical outcomes and adverse events in 136 elderly patients (>75 years of age) with PJI [81]. Out of 136, 25 patients had an adverse drug reaction which led to discontinuation or change of prescription, eight had eventual sepsis, and 13 patients died. The failure-free survival with no event at a follow-up of two years was 61% [81]. It is noteworthy that with infection-free survival reaching 60%, numbers in chronic antibiotic suppression are not far from two-stage revision (true success rate of approximately 65%) [55]. Another study by Sandiford et al. evaluated 26 patients receiving prolonged antibiotic suppression therapy and showed a success rate as high as 84% [82]. In their study, only four patients had an event, two had persistent symptoms and underwent amputation, and two had sepsis successfully managed with intravenous antibiotics [82]. Prolonged suppressive antibiotic therapy seems to be a viable option for selected patients with PJI.

### Last resort: resection arthroplasty (girdlestone) or arthrodesis

For a set of patients who develop a recalcitrant periprosthetic joint infection, surgeons unsuccessfully try DAIR or multiple two revisions. However, nothing seems to work in this particular patient population resulting in difficult treatment decision-making. In this circumstance, there are not many salvage options available. Resection arthroplasty (Girdlestone) [83] and arthrodesis [84] are the salvage procedures available. Girdlestone is hardly used nowadays for knee due to resultant poor outcomes and continuing pain. Nevertheless, results from an infection control standpoint in a recent study by Goldman et al. are rather encouraging [85]. They evaluated 25 knees in which resection arthroplasty was performed over four decades with a mean follow-up of four years. Interestingly, at follow-up, 21 out of 25 knees (84%) were infection-free even though all patients required bracing and assistive devices [86]. More than three decades ago, Bourne et al. investigated 33 Girdlestones in the setting of hip PJI at a mean follow-up of 6.2 years and found similar results [86]. While satisfactory pain relief was achieved in 91% of patients and infection control in 97%, limited functional ability (58%), and leg-length discrepancy were the shortcomings. The authors concluded Girdlestone as a reasonable treatment to salvage repeatedly infected arthroplasty resistant to previous approaches [86]. Arthrodesis is another salvage option that can provide more stability and superior functional outcomes versus resection arthroplasty after failed two-stage knee reimplantation [84]. This surgery can be performed with several techniques: external fixation, intramedullary nail, fixation with cannulated screw, and dual plating with locking compression plate [87–90]. While infection control and eventual rate of union are not different between external fixation and intramedullary nail fixation [87, 90], external fixation is mostly chosen in patients with PJI [89]. Even the fusion can fail in cases with unremitted infection, and treatment involves explantation of the construct, followed by thorough debridement with insertion of antibiotic spacer. On confirmation of the infection control [91], arthrodesis can be attempted again in these patients [92], even though arthrodesis is a complex procedure [93]. In the worst scenario, if local infection spreads systemically increasing the risk of septic shock or severe soft tissue compromise makes the limb unsalvagable, amputation or hip disarticulation could be indicated. Schwartz et al. evaluated the National Inpatient Sample database to determine factors associated with a higher frequency of hip disarticulation and found age under 65 years without private insurance, diabetes with chronic complications, and peripheral vascular disease as the significant risk factors [94].

## Conclusion

In patients with a history of total hip or knee arthroplasty, diligent screening and evaluation are extremely important at the follow-up to achieve an early diagnosis of periprosthetic joint infection. There are many existing PJI diagnostic definitions and markers, the 2013 ICM criteria are the most widely accepted and used criteria for diagnosing an infected arthroplasty. In confirmed infections, two-stage exchange arthroplasty is the treatment of choice in the United States. Encouraging evidence in favor of one-stage revision comes from Europe. This treatment modality is recommended in patients with healthy soft tissues, fair bone stock, and no immunocompromising risk factors. There is a dire need for a randomized controlled trial comparing one- versus two-stage revision, which is already underway in the United States. In a small set of patients in whom no treatment works, and infection recurs after repeated surgeries, salvage procedures are the last resort.

## Conflict of interest statement

The work under consideration for publication: no relevant conflicts of interest. Dr. Higuera reports personal fees and others from KCI, PSI, CD Diagnostics, Cymedica, Ferring Pharmaceuticals, Lyfstone, OREF, Orthofix, Inc., Stryker, Zimmer, American Journal of Orthopedics, Journal of Arthroplasty, Journal of Hip Surgery, Journal of Knee Surgery, American Association of Hip and Knee Surgeons, Mid-American Orthopaedic Association, Musculoskeletal Infection Society, and the work submitted outside. Dr. Pannu and Dr. Villa have nothing to disclose.

## Funding

This research did not receive any specific funding.

## Ethical approval

Ethical approval was not required.

## Informed consent

This article does not contain any studies involving human subjects.

## Author contributions

*Tejbir S. Pannu*: Conception/design of the work, writing, revising, final approval of the manuscript.

*Jesus M. Villa*: Conception/design of the work, writing, revising, final approval of the manuscript.

*Carlos A. Higuera*: Conception/design of the work, writing, revising, final approval of the manuscript.
